# Sandwich-Structured Coating for Ultraviolet Protection and Thermal Management Applications

**DOI:** 10.1021/acsomega.5c09676

**Published:** 2025-11-07

**Authors:** Ugur Kartal, Metin Yurddaskal

**Affiliations:** † The Graduate School of Natural and Applied Sciences, 37508 Dokuz Eylul University, 35390 Izmir, Turkey; ‡ Department of Materials Science and Engineering, Izmir Institute of Technology, 35433 Izmir, Turkey; § Department of Metallurgical and Materials Engineering, Dokuz Eylul University, 35390 Izmir, Turkey; ∥ Center for Fabrication and Application of Electronic Materials, Dokuz Eylul University, 35390 Izmir, Turkey; ⊥ Department of Nanoscience and Nanoengineering, Dokuz Eylul University, 35390 Izmir, Turkey

## Abstract

During the day, exposure to UV radiation poses risks to human health, while managing heat exchange is important for comfort in protective textiles. Recently, infrared-reflective materials have attracted attention, particularly for reducing the infrared transmission and moderating the thermal emission. In this study, titanium dioxide/copper–aluminum/titanium dioxide (TiO_2_/Cu–Al/TiO_2_, TCAT) sandwich-structured coatings were deposited on polyester fabric using magnetron sputtering. Deposition times (40 and 90 s) were varied to adjust Al and Cu layer thicknesses between 20 and 55 nm, and the resulting films were characterized by X-ray diffraction, scanning electron microscopy, X-ray photoelectron spectroscopy, UV–vis spectroscopy, and thermal imaging. The coatings retained moderate visible transmittance on glass, whereas on woven polyester fabric, they formed an optically dense barrier with near-zero UV transmittance, suppressing light penetration across the visible to near-infrared (VIS–NIR) range. In conjunction with the emissivity-aware interpretation of thermography, these results substantiate substrate-independent UV shielding and optical/thermal barrier behavior of the TCAT multilayer. Thermal imaging qualitatively indicated reduced apparent surface temperature for coated fabrics compared to uncoated ones, suggesting partial thermal shielding. These results demonstrate the feasibility of integrating multifunctional coatings into daily-use polyester textiles, offering effective UV protection and the potential for thermal management in protective applications.

## Graphical abstract

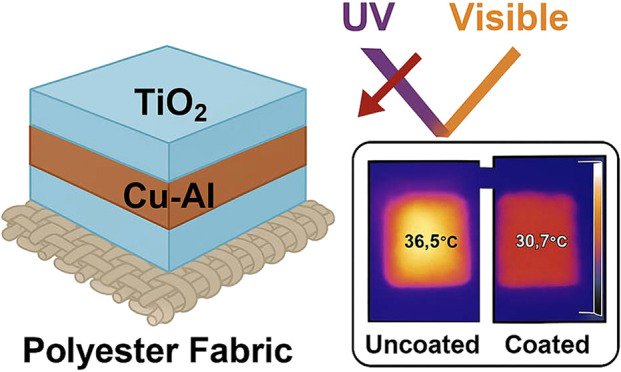

## 1 Introduction

Sandwich-structured coating technology has emerged as a transformative method for enhancing the durability and functionality of materials.^1–5^ By leveraging a multilayered structure, this technology offers superior protection against environmental factors, mechanical wear, and specific spectral requirements, making it an indispensable solution for ultraviolet (UV) protection and thermal management applications.^6–8^ Multilayered or sandwich-structured coatings are effective UV protection technologies that have become a research hotspot, demonstrating the versatility and effectiveness of this innovative approach. These structures have been used to protect against UV/IR waves by absorbing or reflecting UV/IR light and transmitting visible light thanks to an optically active interface layer. Prolonged exposure to UV radiation can cause degradation in materials, reducing their functional lifespan and performance.^9^ Additionally, the harmful effects of UV radiation on human health emphasize the importance of developing protective solutions for everyday products.^10^ Some research has shown that using a highly UV-reflective layer to reduce the harmful effects of UV absorption is an effective strategy.^11^ In contrast, the need for thermal management has grown in fields such as military and personal protection, where thermal emissions detected by thermal imaging devices must be minimized.^12,13^ By reflecting and attenuating IR wavelengths, multilayer coatings can contribute to lowering apparent thermal emission under certain conditions, which may be relevant for protective textile thermal management rather than strict stealth applications.^14^ With the advancement of modern military science and technology, the development of camouflage technologies has been of continued research interest. IR camouflage technology has been studied since the 1940s and is used to hide or camouflage objects to avoid detection by IR cameras.^15^ To minimize detectability, the thermal IR emitted by a target should be matched as closely as possible to the environmental background.^16^ This has usually been achieved by reducing or modifying the IR radiation characteristics. This may involve modifying the IR radiation characteristics of the target or manipulating the pathways of the IR radiation. More advanced thermal management technology has attracted enormous attention around the world.^17^ Multispectral compatibility, integrated design, and intelligent adaptation are the current trends in camouflage technology development.^17^ With the advancement of multispectral detection technology, camouflage with a single frequency band is no longer sufficient to meet complex requirements.^18^

Recently, a novel sandwich-structured coating technology has shown a wide range of applications in thermal management technology due to its unique structure and excellent optoelectronic properties.^19^ In addition, the surface plasmon effect of sandwich-structured coatings for thermal management applications is attracting considerable attention from researchers.^20^ The placement of a thin metal layer between two insulating layers provides a highly effective design for the utilization of the surface plasmon effect.^21^ These dielectric-metal-dielectric sandwich-structured coating systems can offer to adjust optical properties in a multispectral range.^22^ In the surface plasmon phenomenon, the light coming to the metal surface at a certain wavelength interacts with the free electrons of the metal, and the intensity of the reflected light decreases by creating resonance.^23^ In order to achieve this effect, the metal, such as gold (Au), silver (Ag), copper (Cu), and aluminum (Al), used must have a very high free electron capability, and the thickness of the metal film must be much smaller than the wavelength of the incident light.^24^ It has been presented by various researchers in the literature that the dielectric-metal-dielectric layer formed between 5 and 40 nm offers the lowest IR transmittance in the IR region and high transmittance in the visible region, thanks to the high electron mobility surface plasmon effect and Pauli exclusion principle.^25,26^

In this work, we focus on UV shielding and optical/thermal barrier behavior on textiles and explicitly compare glass and woven polyester fabric to assess the substrate-independent performance of the titanium dioxide/copper–aluminum/titanium dioxide (TiO_2_/Cu–Al/TiO_2_, TCAT) architecture. In this study, TCAT was deposited onto glass and polyester fabric through radio frequency (RF) magnetron sputtering for different coating durations to fabricate a multilayered dielectric-metal-dielectric sandwich-structured coating. The significance of this study lies in addressing two key challenges: protecting materials from the harmful effects of UV radiation and fulfilling the demand for thermal management capabilities in specialized applications. It is aimed at investigating how sandwich-structured coating technology can meet these challenges. By combining advanced materials science techniques and innovative design, this study examines the ability of these coatings to provide effective UV protection and thermal management, exploring their potential for diverse real-world applications because the produced TCAT-coated fabrics have potential applications for protective textiles and thermal management, as they have UV protection and thermal management properties. The TCAT combination offers a structure optimized for properties such as optical reflection, low IR transmission, and high thermal insulation. This structure is more economical than more expensive metals such as Au and Ag and provides advantages with its durability and environmental stability. The combination of Cu and Al provides high electrical conductivity, mechanical strength, and good optical performance, while the TiO_2_ layers provide strong optical reflection properties. The surface morphology of the films was analyzed using a scanning electron microscope (SEM). X-ray diffraction (XRD) analysis was conducted to investigate the crystal structure of the films. The chemical, optical, and thermal properties of the films were also examined by using X-ray photoelectron spectroscopy (XPS), UV–vis spectroscopy, and a thermal camera.

## 2 Experimental Procedure

### 2.1 Production of Films

For RF magnetron sputtering, TiO_2_ (99.99%), Al (99.99%), and Cu (99.99%) target materials with a diameter of 2″ and a thickness of 0.25″ were purchased from Kurt Lesker (USA). Technical specifications of these materials are given in Table 1.

**Table 1:**
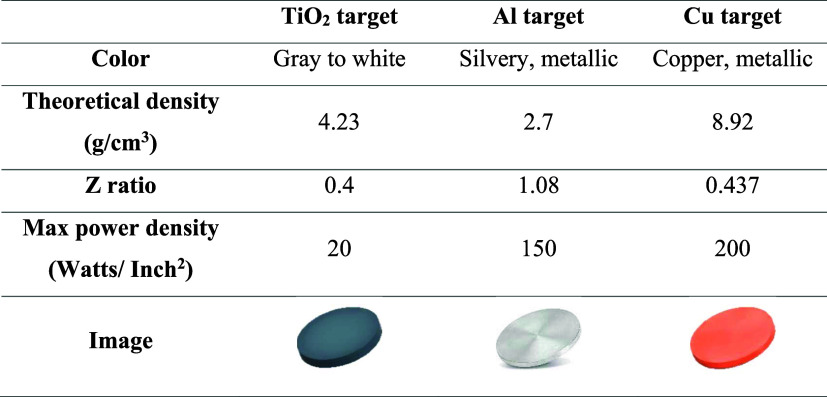
Technical Properties of Target Materials

Commercially purchased target materials were used to deposit films in the RF magnetron sputtering system. Lamella glass substrates were cleaned by a standard procedure with ethanol (C_2_H_5_OH) and distilled water before being placed in the sputtering chamber. Precleaned glass substrates and polyester fabrics cut in appropriate sizes were exposed to an oxygen plasma (Femto Science, CUTE). Plasma cleaning process was performed using oxygen gas at 0.07 Torr pressure and 22 sccm gas flow rate for 3 min with 80 W energy.

The chamber of the RF Magnetron Sputter device (Nanovak, NVTH-350) was placed in vacuum after the substrate materials to be coated were placed in the sample holder (298 K temp) with a distance of 10 cm between them and the target material. Details of parameters for all deposition conditions such as temperature, gas flow rates, power, and chamber gas pressure are shown in Table 2.

**Table 2:** RF Magnetron Sputtering Parameters and Conditions

**target material**	TiO_2_	Cu	Al
**RF power (Watt)**	60	30	40
**base vacuum (Torr)**	3–4.5 × 10^–6^	3- 4.5 × 10^–6^	3- 4.5 × 10^–6^
**deposition pressure (Torr)**	1.05 × 10^–2^	1.05 × 10^–2^	1.05 × 10^–2^
**gas flow (sccm)**	31	28	29
**deposition rate** (Å/s)	0.0 – 0.1	5	5

Cu and Al layers were deposited at different times (40–90 s) to examine the effect of coating thickness on optical properties. To enhance the adhesion of the metal Cu layer to the glass substrate, the TiO_2_ layer was first deposited for 1 h. After the formation of Cu- and Al-based metallic coatings, the TiO_2_ layer was again deposited for 1 h on the top to prevent corrosion and oxidation of metallic layers. All samples were named depending on the deposition time applied and are given in Table 3. The image in Table 3 shows an image of the TC5A8T sample before thermal measurement.

**Table 3:**
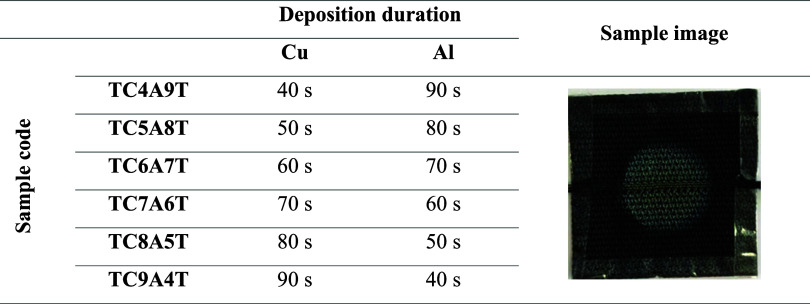
Sample Code of Sandwich-Structured Film Layers, Deposition Times, and Example Visual of TC5A8T Sample

### 2.2 Characterization

After the coatings were deposited on the substrate, XRD measurements were performed. Thermo Scientific’s ARL X’TRA model instrument was used to obtain XRD patterns. Cu–Kα (1.54185 Å) radiation was used at 40 kV and 40 mA to determine the crystal structures of the samples, and scanning was performed between 10 and 80° at a rate of 4°/min at room temperature. SEM device (Zeiss, Gemini) was used to examine the surface morphology of coatings using the secondary electron method at 10–13 kV applied voltage. A Thermo Scientific K-α XPS instrument with a monochromatic Al–Kα (1486.7 eV) X-ray source was used to analyze the chemical presence of sandwich-structured coatings and their successful overlap in the desired order. For this purpose, depth profile analysis was performed on the coatings on the glass surface produced within the scope of this study with a high current ion power of 2000 eV for 40 s periods. The thickness of each of the produced film layers was measured by using the Filmetrics F20 device as a simple method. Optical characterization of the produced samples was carried out with a UV–vis spectrophotometer (SHIMADZU UV-2600i) equipped with an integrated sphere attachment. Thermal imaging of polyester fabrics covered with sandwich-structured coatings was performed with the help of a thermal camera (Fluke, Ti27). For thermal imaging, a hot plate was heated to 42–43 °C in a laboratory with an average room temperature of 26 °C, and the coated fabrics were placed on this plate. Tape was applied to the four corners of the fabric to prevent the fabric fibers from separating. After waiting for 30 s, thermal camera images were taken. Thermal camera measurements provide qualitative indications of apparent temperature differences under our laboratory setup and do not substitute for spectrally resolved 8–14 μm emissivity/reflectance data. The surface temperature measurements were interpreted qualitatively, given that commercial IR cameras typically operate with a fixed emissivity assumption of ∼ 0.9. For surfaces with low emissivity, such as those composed of metals, this assumption can result in a slight underestimation of the apparent temperature.

## 3 Results and Discussion

The SEM images of TCAT-based coatings deposited on glass substrates (produced as a control sample for characterization) with an RF magnetron sputtering system are given in Figure 1. When the images of the produced coatings were examined, it was seen that the films were coated smoothly and homogeneously but had some impurities. The reason for the impurities and heterogeneous zones can be cited as the magnetron sputtering device’s chamber not being fully cleaned prior to production, the films being used as prepared, and not being subjected to heat treatment.

**Figure 1:**
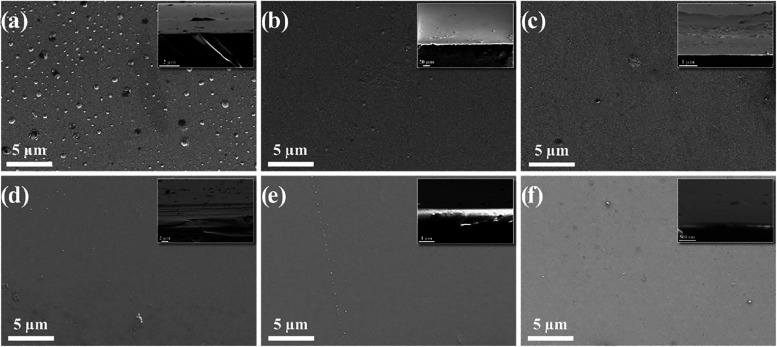
Top-view SEM images of the TCAT samples deposited on glass substrates: (a) TC4A9T, (b) TC5A8T, (c) TC6A7T, (d) TC7A6T, (e) TC8A5T, and (f) TC9A4T (side views of samples are shown in the top-right insets).

XRD patterns of untreated and TCAT-coated glass substrates sputtered for different times are given in Figure 2. Since no heat treatment was applied to the deposited substrates prior to XRD analysis, the crystalline peak of the amorphous glass substrates and TiO_2_ remaining in an amorphous form were not observed. This may be due to the high temperature required for the crystallization of TiO_2_ films. Furthermore, TiO_2_ films are mostly in an amorphous form when deposited at room temperature. The peaks at 43.4, 50.3, and 74.1°, which are associated with the following planes (111), (200), and (220), respectively, show good agreement with the ICDD card No. 01-085-1326 of Cu.^27^ On the other hand, the peaks at 38.4, 44.7, 65.1, and 78.2° match with the planes (111), (200), (220), and (311), respectively, as reported in the ICDD card No. 00-004-0787 of Al.^28^

**Figure 2:**
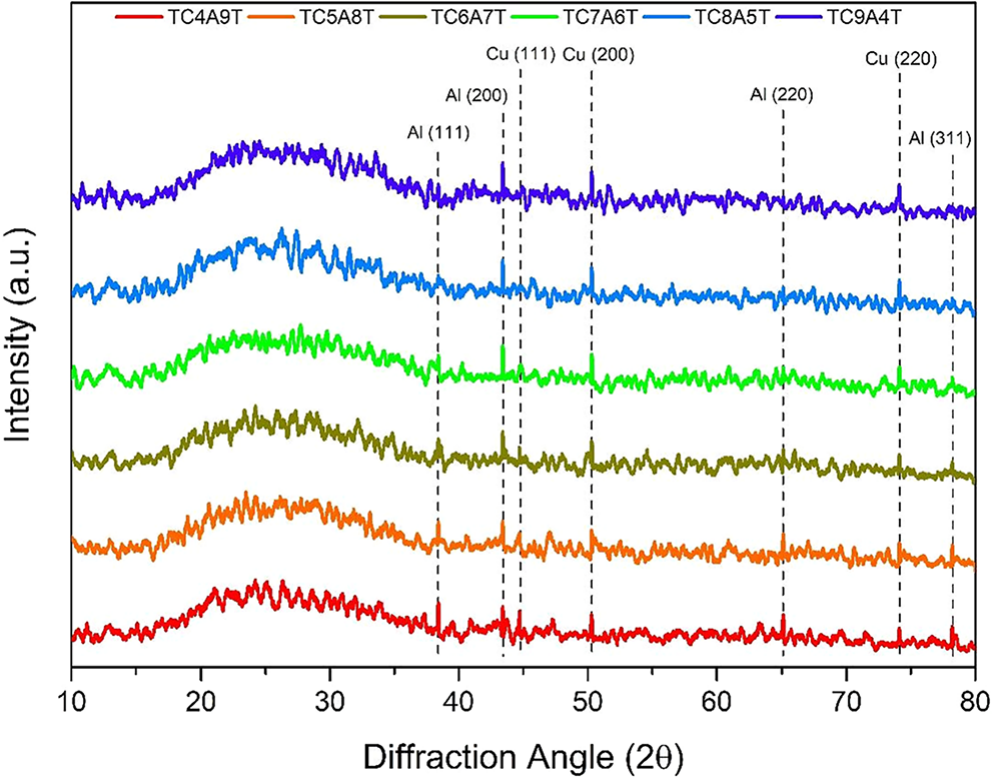
XRD patterns of the TCAT-coated glass substrates.

The wide survey spectra of TiO_2_, Cu, and Al-based monolayer films (identified by element name and numbers from 4 to 9 corresponding to 40 to 90 s) recorded over a binding energy range of 0 to 1350 eV were utilized for elemental mapping (Figure 3). The spectra of all samples displayed a typical carbon peak, C 1s, at 284.80 eV serving as the reference peak.^29^ Cu monolayer-coated glass featured peaks for Cu 2p, O 1s, C 1s, and Na 1s, while Al monolayer-coated glass displayed peaks for Al 2p, O 1s, C 1s, and Na 1s in its spectrum.^30^ Since soda-lime glass served as the substrate, it was concluded that the Na 1s and O 1s peaks come from it.^31^ The spectra of glass with a TiO_2_ monolayer showed peaks at Ti 2p, C 1s, and O 1s.^32^ The lack of extra fundamental peaks in the spectra indicates that the coatings do not contain any foreign substances.

**Figure 3:**
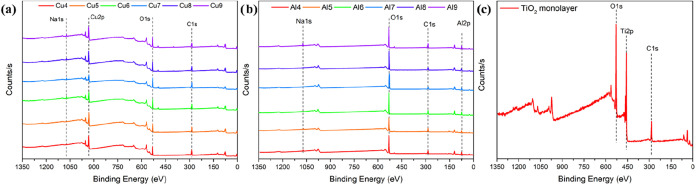
Wide survey spectra of Cu-, Al-, and TiO_2_-based monolayer films on glass substrates: (a) Cu layers, (b) Al layers, and (c) TiO_2_ monolayer.

Figure 4 shows that Ti 2p and O 1s can be observed up to 120 s during the ion etching process, while Cu 2p and Al 2p are absent. These components are present due to the TiO_2_ layer applied on top to protect the metal layers from oxidation and corrosion. The presence of metallic layers is clearly seen due to the increase in atomic weight percentages of Cu 2p and Al 2p from 120 to 200 s. In addition, after 120 s of ion etching, the TiO_2_ layer disappears, and the metallic layer is exposed, which is evidenced by the decrease in the Ti 2p and O 1s peaks observed here. After 280 s of ion etching, the atomic weight percentages of Ti 2p and O 1s increased due to the underlying TiO_2_ layer added to enhance metal adhesion to the substrate, while the atomic weight percentages of Cu 2p and Al 2p decreased again. The absence of the Cu 2p peak and the prominent O 1s peak in the TC4A9T sample is due to the ion etching reaching the glass substrate rapidly by completely etching the coating. The high O 1s peak observed on the glass substrate is due to the O element in SiO_2_. Thus, it was seen by depth profile analysis that the desired sandwich structure coating was successfully applied.

**Figure 4:**
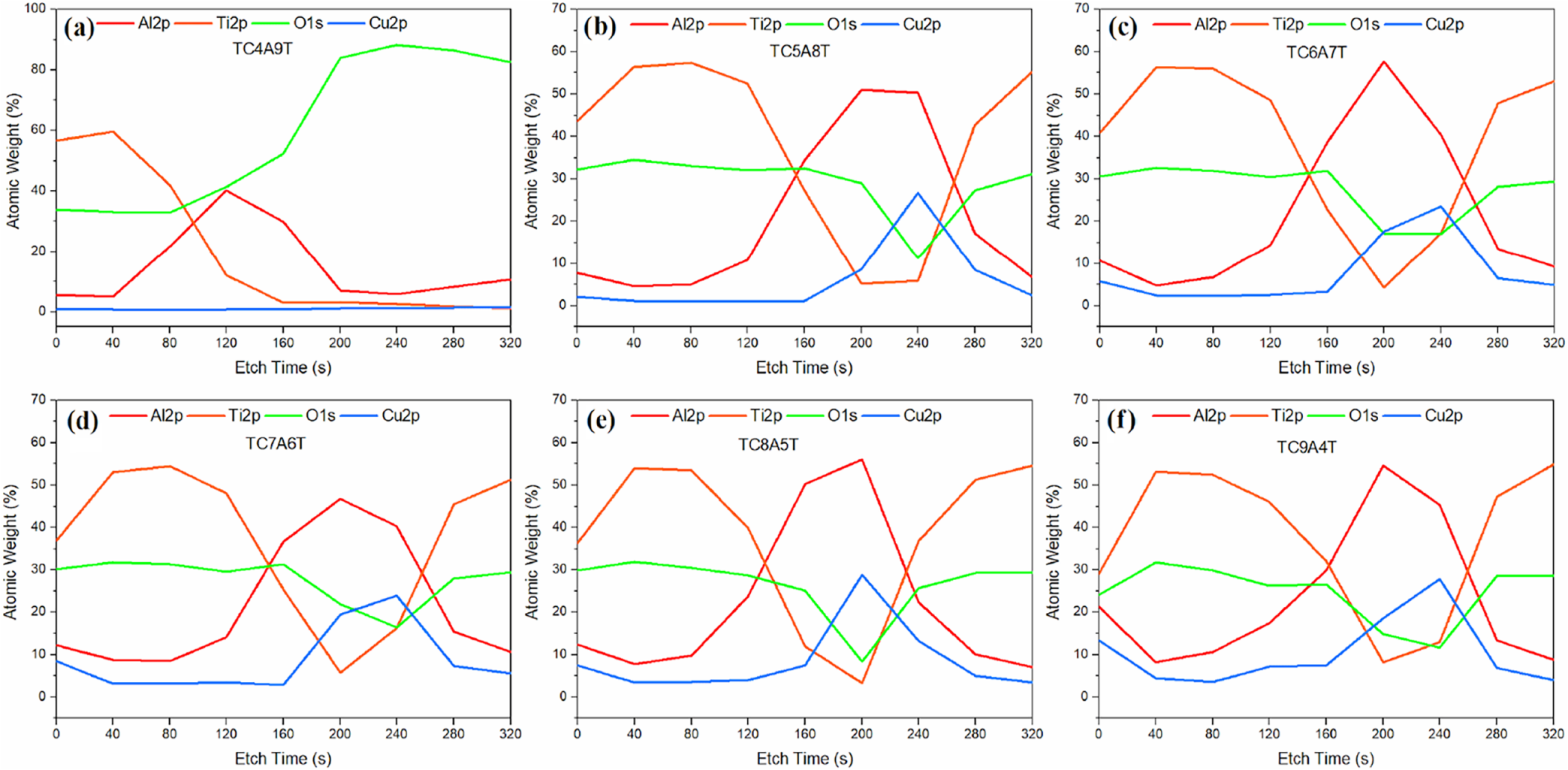
Depth profile analysis of TCAT-based coatings: (a) TC4A9T, (b) TC5A8T, (c) TC6A7T, (d) TC7A6T, (e) TC8A5T, and (f) TC9A4T.

Cu and Al single layers were produced for control purposes with the same parameters as those of the sandwich structure. Each sample was produced 3 times, and 3 measurements were taken from all samples. According to the results given in Figure 5, the Cu and Al layer thicknesses were in the range of 20–55 nm (RF deposition rate 5 Å/s).

**Figure 5:**
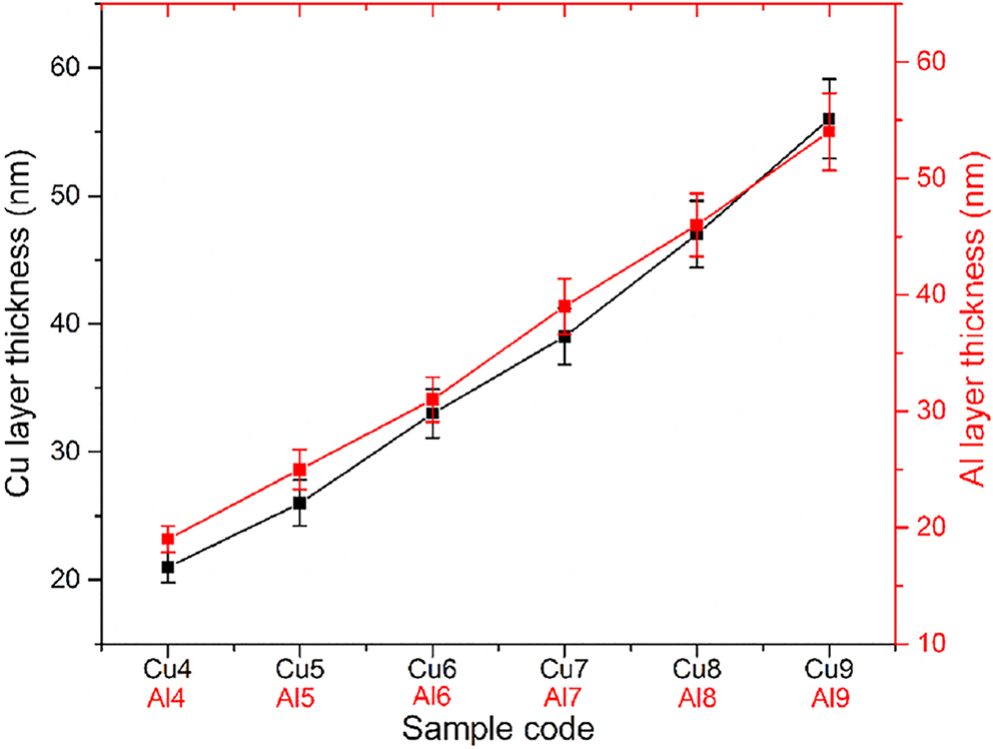
Layer thicknesses of Cu- and Al-monolayered control samples.

When the incident light is irradiated to the metal surface, the free electrons on the metal surface are rapidly excited, and the photons in the incident light vibrate at the same frequency, causing resonance on the metal surface, resulting in surface plasmon resonance. Meanwhile, the intensity of the optical field at the interface increases significantly, and strong resonant absorption or scattering is observed in the spectrum.^33^ Thus, the spectrum with changing absorbance and transmittance properties is observed. After the TCAT-based coating, the UV transmittance of the glass substrates decreased significantly in the UV region, especially in the UV-B range (290–320 nm), as shown in Figure 6a. When the transmittance properties of the coatings in the UV-A region were examined, it was observed that the UV transmittance is almost zero. This indicates that the thickness of the Cu and Al layers has a significant effect on UV transmittance. In the UV-B region, the lowest optimum UV transmittance was provided by the TC4A9T sample. As shown in Figure 6a, the UV transmittance of the coated samples is almost zero. In the visible region, all coatings retained ∼60–70% (Figure 6b) transmittance. In the near-infrared (NIR), the transmittance remained relatively high (∼70%) with modest thickness-dependent variations. These trends are consistent with carrier-concentration effects in ultrathin metal-dielectric stacks and indicate limited NIR blocking, while the LWIR (8–14 μm) behavior was not spectrally quantified in this study. The variation of the film’s transmittance in the near-IR (NIR) region as a function of metal layer combinations is shown in Figure 6c. Unlike the film’s transmittance in the visible light region, the film’s optical transmittance behavior in the NIR region varies around 70%. The lowest IR transmittance was observed in the TC4A9T sample. Generally, the optical property of TCAT-based coatings in the NIR region depends on the carrier concentration. Since increasing carrier concentration provides shorter plasma wavelengths, films with higher carrier concentrations show lower transmittance in the NIR region.^34–36^ Depending on the parameters, Cu and Al layers that increase and decrease simultaneously showed an average IR transmittance of 70%. Furthermore, in order to verify that this optical-barrier behavior also holds on flexible substrates, UV–VIS–NIR reflectance and transmittance spectra of TCAT-coated woven polyester fabrics were measured (Figure 7).

**Figure 6:**
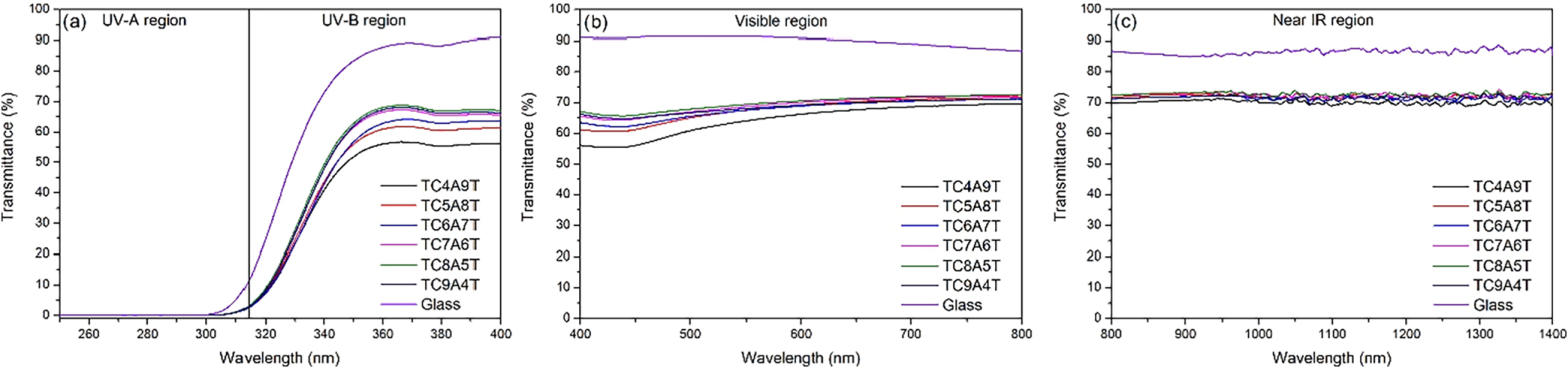
Optical transmittance spectra of TCAT-based films deposited on a glass surface: (a) UV region, (b) Vis region, and (c) NIR region.

**Figure 7:**
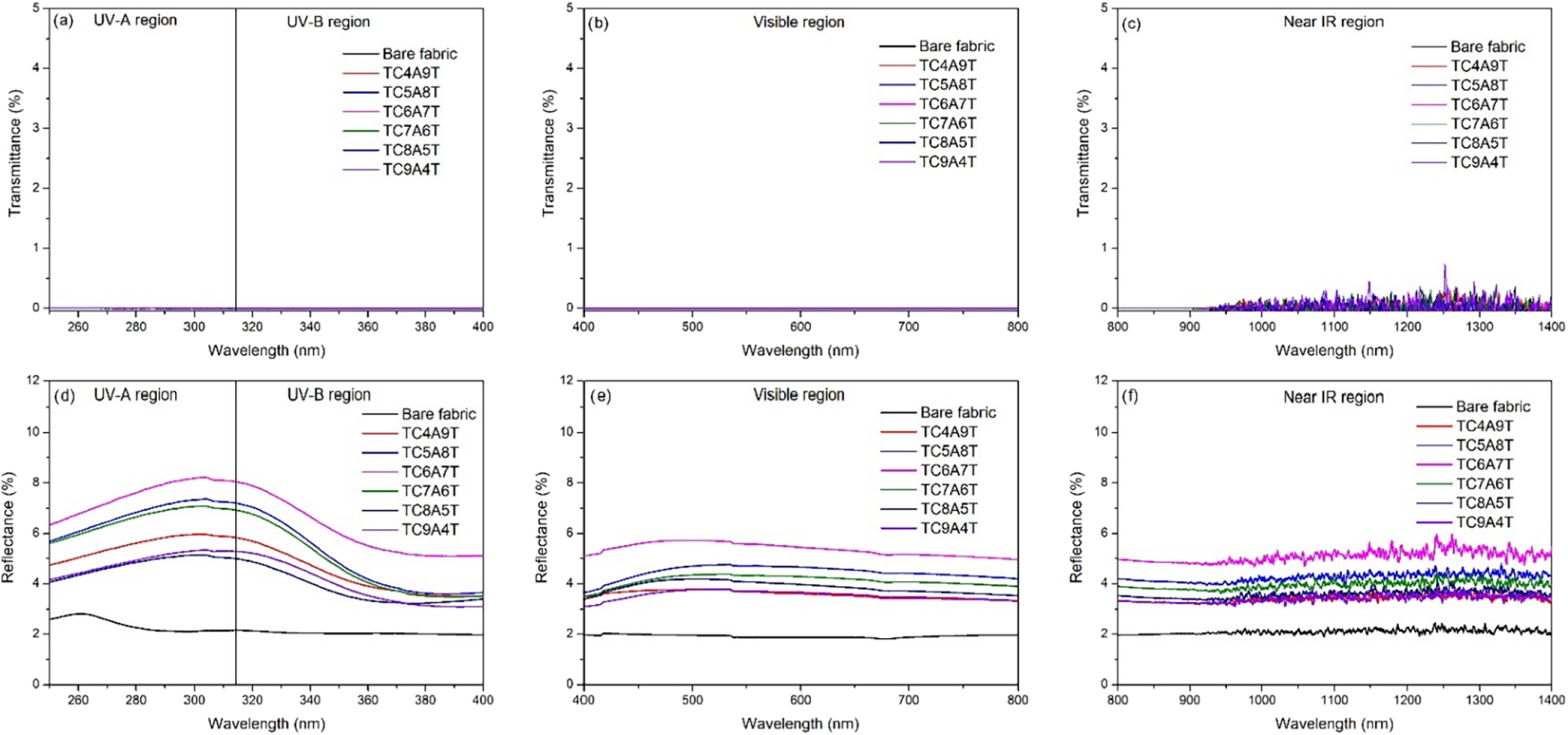
UV–VIS–NIR reflectance and transmittance spectra of TCAT (TiO_2_–(Cu/Al)–TiO_2_) coatings deposited on woven polyester fabrics.

The Pauli exclusion principle plays a key role in shaping the electronic structure of metals, thereby influencing their interaction with infrared (IR) radiation and the corresponding transmittance. In Al and Cu, the conduction bands are relatively filled, meaning that electronic states are largely occupied; as a result, photons in the IR range cannot be readily absorbed unless their energies correspond to available transitions between states. Consequently, IR photons are more likely to be reflected or weakly absorbed rather than transmitted through these metallic layers. The visible and near-infrared optical responses observed in this study are consistent with expectations for ultrathin Cu–Al layers embedded within TiO_2_ spacers, where free-carrier effects and interfacial interference modulate both transmittance and reflectance. Furthermore, the interfaces formed between the metallic and dielectric layers enhance the reflection and absorption pathways, thereby contributing to the overall reduction of IR transmission in the coating.

It is evident from Figure 7 that both bare and TCAT-coated fabrics exhibit negligible UV transmittance (<1%), a consequence of their dense and scattering textile texture. It is crucial to note that the TCAT stack maintains near-zero UV transmittance while systematically increasing hemispherical reflectance across the UV–VIS–NIR range. This indicates that incident radiation is predominantly managed by surface reflection and intralayer absorption rather than penetrating the textile volume. In summary, the coating forms a continuous, optically dense barrier at the fabric–air interface. This barrier-type response on fabric is fully consistent with the behavior previously observed on glass, thus confirming the substrate-independent UV shielding of the TCAT architecture.

The SEM images of fabrics coated with a TCAT-based coating are shown in Figure 8. Upon examination of these images, it is clear that the polyester fabric fibers were coated in all samples. The EDX results given in Figure 9 also support the successful application of the coatings, and a homogeneous distribution is observed in the element maps. However, the coating seemed to be inconsistent in certain areas. This nonuniformity may be due to the rough fiber structure of the substrate material and the relatively short duration of the coating process.^37^

**Figure 8:**
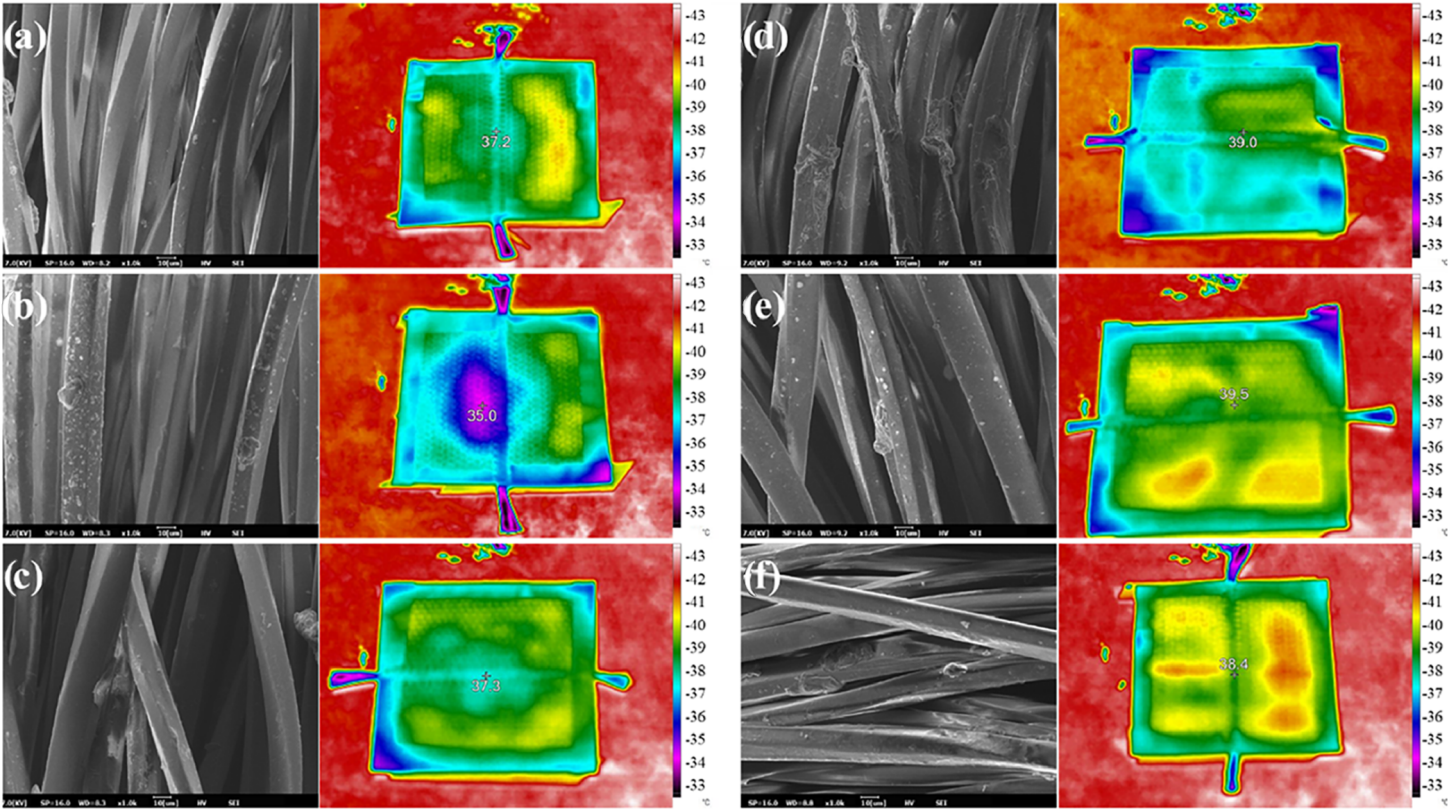
SEM images (left side) and thermal camera images (right side) of TCAT-coated polyester fabrics: (a) TC4A9T, (b) TC5A8T, (c) TC6A7T, (d) TC7A6T, (e) TC8A5T, and (f) TC9A4T.

**Figure 9:**
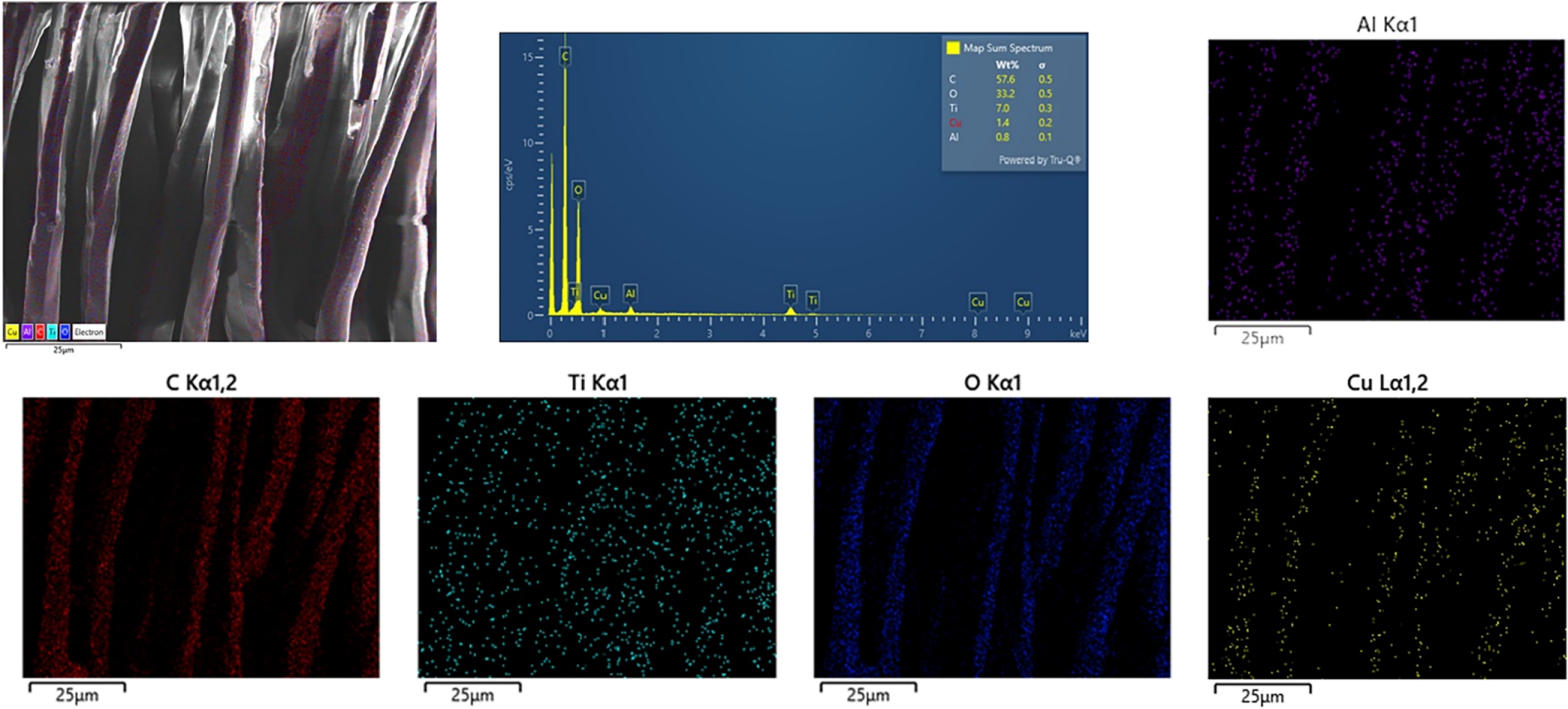
EDX results of the TC5A8T sample.

The thermographic properties of TCAT-deposited polyester fabrics were examined, and thermal camera images are given in Figure 8. These images correspond to fabric specimens with dimensions of approximately 2 cm × 2 cm, enabling size estimation even though the images themselves do not contain a scale bar. The set temperature of the environment where the photos were taken can be seen as red areas (42 °C) in the images. This temperature is more than enough to examine the studies of preventing the reflection of human body temperature, comparable to human body temperature (∼36.5–37 °C), making it relevant for evaluating textile thermal management.^38^ When all samples are examined, it is seen that the coated region in the sample named TC5A8T has the lowest temperature value at 35 °C. The reason for this is the different thickness values resulting from the deposition of Cu and Al at different times. The localized surface plasmon formed in Cu and Al layers of different thicknesses and between these layers has created variable effects in the direction of IR light.^39^ The lower thickness of the Cu layer and higher thickness of the Al layer in the TC5A8T sample show that the localized surface resonance was the best in this sample in terms of thermographic performance. Under our laboratory conditions, coated fabrics exhibited a reduced apparent surface temperature relative to uncoated controls (e.g., TC5A8T), which suggests partial thermal shielding and indicates the potential for thermal management in textiles. The IR camera operates under the assumption of an emissivity of approximately 0.9, implying that the apparent temperature is emissivity dependent. It has been established that the outer TCAT interface is identical on glass and polyester fabric. Thus, any reduction in the emissivity would have a similar effect on both surfaces. Consequently, the observed thermographic contrast is attributed to the coating’s intrinsic radiative and optical properties rather than a substrate artifact.

## 4 Conclusions

This study investigated TiO_2_/Cu–Al/TiO_2_ (TCAT) multilayer dielectric-metal-dielectric coatings produced by RF magnetron sputtering on glass substrates and polyester fabrics. Structural and surface analyses confirmed the successful fabrication of homogeneous, well-defined sandwich architectures with amorphous TiO_2_ and crystalline Cu–Al layers. XPS depth profiling further validated the integrity of the multilayer structure. The coatings exhibited markedly reduced UV transmittance while retaining moderate visible transparency. Thermal imaging under controlled conditions also indicated reduced apparent surface temperature for coated fabrics relative to uncoated controls, suggesting potential for thermal management. The optical response was influenced by metallic layer thickness, highlighting the role of free-carrier and interfacial effects and the tunability of the design. It is imperative to emphasize that the assertion pertaining to the thermal management of the coating is contingent upon its capacity to function as an optical/thermal barrier, thereby diminishing light penetration and radiative heat transfer. The observation that the emissivities of glass and polyester fabric are comparable lends further support to the hypothesis that the observed temperature trends are a consequence of the intrinsic radiative behavior of the TCAT surface. The fabric-based UV–VIS–NIR spectra corroborate near-zero UV transmittance and enhanced reflectance on textiles, consistent with the optical behavior observed on glass. In conjunction with the emissivity-aware thermographic analysis, these results substantiate substrate-independent UV shielding and optical/thermal barrier performance of the TCAT coatings. Overall, these findings demonstrate the feasibility of integrating TCAT coatings into daily-use polyester textiles, providing effective UV shielding and preliminary evidence of thermal management capability. Future research should explore alternative metallic layers and multilayer combinations to enhance performance, as well as systematic testing under varying ambient temperatures and assessments of surface wettability to broaden functional applications.

## References

[1] Birman V., Kardomateas G. A. (2018). Review
of Current Trends in Research and Applications
of Sandwich Structures. Composites, Part B.

[2] Zhang Y., Wu H., Guo S. (2022). Sandwich-Structured
Surface Coating of a Silver-Decorated
Electrospun Thermoplastic Polyurethane Fibrous Film for Excellent
Electromagnetic Interference Shielding with Low Reflectivity and Favorable
Durability. ACS Appl. Mater. Interfaces.

[3] Le J., Lv F., Lin J., Wu Y., Ren Z., Zhang Q., Dong S., Luo J., Shi J., Chen R., Hong Z., Huang Y. (2024). Novel Sandwich-Structured
Flexible
Composite Films with Enhanced Piezoelectric Performance. ACS Appl. Mater. Interfaces.

[4] Yi Z., Wang Z., Nian W., Wang T., Chen H., Cheng Z. (2021). High Energy Storage Density of Sandwich-Structured Na0.5Bi0.5TiO3/PVDF
Nanocomposites Enhanced by Optimizing the Dimensions of Fillers. ACS Appl. Energy Mater..

[5] Ding D., Maeyoshi Y., Kubota M., Wakasugi J., Kanamura K., Abe H. (2019). A Facile Way to Synthesize Carbon-Coated LiMn0.7Fe0.3PO4/Reduced
Graphene Oxide Sandwich-Structured Composite for Lithium-Ion Batteries. ACS Appl. Energy Mater..

[6] Subramaniyan M., Karuppan S., Radhakrishnan K., Rajesh Kumar R., Saravana Kumar K. (2022). Investigation of Wear Properties of 3D-Printed PLA
Components Using Sandwich Structure – A Review. Mater. Today Proc..

[7] Militký J., Křemenáková D., Venkataraman M., Večerník J., Martínková L., Marek J. (2021). Sandwich Structures Reflecting Thermal Radiation Produced by the
Human Body. Polymers.

[8] Han Z., Chen R., Li J., Guo S. (2025). Enhancement of Radar-Infrared
Stealth Performance of EPDM-Based Composites through the Asymmetric
Sandwich Structural Construction. Compos. Sci.
Technol..

[9] Andrady A. L., Heikkilä A. M., Pandey K. K., Bruckman L. S., White C. C., Zhu M., Zhu L. (2023). Effects of UV Radiation on Natural and Synthetic Materials. Photochem. Photobiol. Sci..

[10] Neale R. E., Lucas R. M., Byrne S. N., Hollestein L., Rhodes L. E., Yazar S., Young A. R., Berwick M., Ireland R. A., Olsen C. M. (2023). The Effects of Exposure
to Solar
Radiation on Human Health. Photochem. Photobiol.
Sci..

[11] Hanif M. A., Shin H., Chun D., Kim H. G., Kwac L. K., Han S. W., Kang S. S., Kim Y. S. (2024). Development
of Highly
Ultraviolet-Protective Polypropylene/TiO2 Nonwoven Fiber. J. Compos. Sci..

[12] Wu Y., Tan S., Zhao Y., Liang L., Zhou M., Ji G. (2023). Broadband
Multispectral Compatible Absorbers for Radar, Infrared and Visible
Stealth Application. Prog. Mater. Sci..

[13] Shirke N., Ghase V., Jamdar V. (2024). Recent Advances
in Stealth Coating. Polym. Bull..

[14] Hou K., Ma H., Zhao H., Li X., Wang J., Cai Z. (2023). Fabrication
of Sandwich-Structured Infrared Camouflaged and Flexible Anti-Aging
Composite for Thermal Management. Ceram. Int..

[15] Lu Z., Shen X., Li Z., Jia Q. (2024). Flexible Sandwich Structure
MXene-Aramid Nanofiber-MXene Film for Adjusting Infrared Camouflage
and Multifunctional Application. Compos. Struct..

[16] Hu R., Xi W., Liu Y., Tang K., Song J., Luo X., Wu J., Qiu C. W. (2021). Thermal Camouflaging Metamaterials. Mater. Today.

[17] Zhou Y., Rather L. J., Yu K., Yang M., Lu M., Li Q. (2024). Research Progress and Recent Advances in Development
and Applications
of Infrared Stealth Materials: A Comprehensive Review. Laser Photonics Rev..

[18] Zhao Y., Ji G. (2022). Multi-Spectrum Bands
Compatibility: New Trends in Stealth Materials
Research. Sci. China Mater..

[19] Wang L., Wang W., Wang L., Liu G., Ge C., Xu K., Wang B., Liu T. (2024). Flexible and
Transparent Visible-Infrared-Compatible
Stealth Film Based on ITO/Ag/ITO Configuration. J. Opt..

[20] Li D., Chen Q., Huang J., Xu H., Lu Y., Song W. (2022). Scalable-Manufactured
Metamaterials for Simultaneous Visible Transmission,
Infrared Reflection, and Microwave Absorption. ACS Appl. Mater. Interfaces.

[21] Willey R. R., Stenzel O. (2023). Designing Optical Coatings with Incorporated Thin Metal
Films. Coatings.

[22] Liu Y., Ma W. Z., Wu Y. C., Meng D., Dou C., Cheng Y. Y., Chen Y. S., Liu J., Gu Y. (2023). A Metamaterial
Absorber with a Multi-Layer Metal–Dielectric Grating Structure
from Visible to near-Infrared. Opt. Commun..

[23] Roques-Carmes C., Kooi S. E., Yang Y., Rivera N., Keathley P. D., Joannopoulos J. D., Johnson S. G., Kaminer I., Berggren K. K., Soljačić M. (2023). Free-Electron-Light
Interactions
in Nanophotonics. Appl. Phys. Rev..

[24] Zhang C., Ji C., Park Y.-B., Guo Jay., Zhang L., Ji C., Park C., Guo Y. (2021). Thin-Metal-Film-Based Transparent
Conductors: Material Preparation, Optical Design, and Device Applications. Adv. Opt Mater..

[25] Wu J. Y., An B. L., Dong W., Yang Z., Duan Y. Y. (2024). Design,
Preparation, and Property Analysis of Metal/Dielectric Multilayer
Film with Wavelength Selectivity. J. Phys.:
Condens. Matter.

[26] Zhang B., Gong R., Zhang Y., Li Y., Zhu L. (2023). Recent Progress
in Dielectric/Metal/Dielectric Electrodes for Foldable Light-Emitting
Devices. Nanotechnol. Rev..

[27] Pandiyarajan S., Hsiao P. J., Liao A. H., Ganesan M., Manickaraj S. S. M., Lee C. T., Huang S. T., Chuang H. C. (2021). Influence of Ultrasonic
Combined Supercritical-CO2 Electrodeposition Process on Copper Film
Fabrication: Electrochemical Evaluation. Ultrason.
Sonochem..

[28] Kumar U., Vaghela H. B., Koshy A. M., Swaminathan P. (2024). Low Emissivity
Thin Film Coating on Glass Fiber Reinforced Plastic Used for Cryogenic
Application. J. Mater. Sci.:Mater. Electron..

[29] Ruan Y., Deng B., He D., Chi R. (2021). Synergetic Effect of
Cottonseed Fatty Acid Salt and Nonionic Surfactant NP-4 in the Froth
Flotation of Siliceous-Calcareous Phosphate Rock. Colloids Surf., A.

[30] Fu Z., Huang Y., Zheng Z., Zhang Y., Xu J., Chen S., Zhang H. (2024). Electrochemical
Behavior and Corrosion
Mechanism of Copper Alloys in Coastal Environment Via Al/Ni Doping
Strategy. J. Mater. Eng. Perform..

[31] Roy B., Baier F., Rosin A., Gerdes T., Schafföner S. (2023). Structural
Characterization of the Near-Surface Region of Soda–Lime–Silica
Glass by X-Ray Photoelectron Spectroscopy. Int.
J. Appl. Glass Sci..

[32] Yu Y., Matsunaga M. (2025). Sol–Gel Electrophoretically Deposited TiO2–Multiwalled
Carbon Nanotube–SiO2 Thin-Film Electrode with High Photoelectrochemical
Activity. ACS Omega.

[33] Li M., Li Y., Zou N., Wu J., Bo X., Chu J. (2024). A Linearly
Polarized Light Emission with a Composite Nanowire Grating in Whole
White Band. Phys. Scr..

[34] Wang K. L., Xin Y. Q., Zhao J. F., Song S. M., Chen S. C., Lu Y. B., Sun H. (2018). High Transmittance
in IR Region of
Conductive ITO/AZO Multilayers Deposited by RF Magnetron Sputtering. Ceram. Int..

[35] Yang C., Wöll C. (2017). IR Spectroscopy
Applied to Metal Oxide Surfaces: Adsorbate
Vibrations and Beyond. Adv. Phys. X.

[36] Jin Y., Jeong Y., Yu K. (2023). Infrared-Reflective
Transparent Hyperbolic
Metamaterials for Use in Radiative Cooling Windows. Adv. Funct Mater..

[37] Barakhovskaia E., Apicella L., Glushchuk A., Minetti C., Iorio C. S. (2022). A Fast
Methodology to Assess the Quality of Coatings on Rough 3D Surfaces. Diamond Relat. Mater..

[38] Zhang H., Zhang Y., Liu Y., Zhang Q., Zhang Y., Shan Z., Liu X., Zhao J., Li G., Yang D. P. (2023). Self-Assembled and Multilayer-Overlapped ESM-PDA@rGO
Nanofilm-Based Flexible Wearable Sensor for Real-Time Body Temperature
Monitoring. ACS Appl. Mater. Interfaces.

[39] Liao J., Zhan Y., Liu Q., Hong R., Tao C., Wang Q., Lin H., Han Z., Zhang D. (2021). Tunable Surface
Plasmon Resonance of Al-Cu Bimetallic Nanoparticles Thin Films Induced
by Pulsed-Laser. Appl. Surf. Sci..

